# Efficacy of 0.05% Cyclosporine-A eye drops (II) and 3% Diquafosol ophthalmic solution in the treatment of dry eye after cataract surgery

**DOI:** 10.12669/pjms.40.11.10607

**Published:** 2024-12

**Authors:** Hongwei Lu, Shuangmei Zhang, Chenjun Shao, Pengfei Chen, Minting Ma, Yuhua Hao

**Affiliations:** 1 Hongwei Lu Department of Ophthalmology, Fourth Hospital of Hebei Medical University, Shijiazhuang, Hebei Province 050000, P.R. China; 2 Shuangmei Zhang Department of Ophthalmology, Fourth Hospital of Hebei Medical University, Shijiazhuang, Hebei Province 050000, P.R. China; 3 Chenjun Shao Department of Ophthalmology, Fourth Hospital of Hebei Medical University, Shijiazhuang, Hebei Province 050000, P.R. China; 4 Pengfei Chen Department of Ophthalmology, Fourth Hospital of Hebei Medical University, Shijiazhuang, Hebei Province 050000, P.R. China; 5 Minting Ma Department of Oncology, Fourth Hospital of Hebei Medical University, Shijiazhuang, Hebei Province 050000, P.R. China; 6 Yuhua Hao Department of Ophthalmology, Fourth Hospital of Hebei Medical University, Shijiazhuang, Hebei Province 050000, P.R. China

**Keywords:** 0.05% Cyclosporine-A eye drops (II), 3% Diquafosol ophthalmic solution, Dry eye, Cataract surgery

## Abstract

**Objectives:**

To compare the efficacy of 0.05% cyclosporine A (CsA) eye drops (II) and 3% Diquafosol ophthalmic solution (DQS) in the treatment of dry eye (DE) after cataract surgery (CS).

**Methods:**

Clinical data of 123 patients with DE after CS treated at the Fourth Hospital of Hebei Medical University from June 2022 to August 2023 were retrospectively analyzed. Patients were divided into three groups based on the treatment: Conventional group (tobramycin & dexamethasone eye drops combined with pranoprofen eye drops, n=41), DQS group (3% DQS based on the conventional treatment, n=42), and CsA group (0.05% CsA eye drops(II) based on the conventional treatment, n=40). The therapeutic effects; Schirmer I test (SIt), tear film breakup time (TBUT), levels of serum inflammatory factors, and tear cytokine levels before and after treatment were compared between the groups.

**Results:**

Chief complaint score, conjunctival congestion score, corneal fluorescein staining score, and ocular surface disease index score of the DQS group and the CsA groups were significantly lower than those of the conventional group (*P*<0.05). After treatment, the improvement in SIt, TBUT, serum inflammatory factors, and tear cytokine levels in the DQS group and the CsA group was significantly better than that in the conventional group (*P*<0.05). However, these indexes were comparable in the DQS and the CsA group (*P*>0.05).

**Conclusions:**

Compared to the conventional treatment alone, the addition of 3% DQS or 0.05% CsA eye drops (II) to the conventional treatment both are effective and might more effectively alleviate DE in patients undergoing CS.

## INTRODUCTION

Cataract surgery (CS), one of the most common ophthalmic surgeries,^1^ is associated with high (20 -50%) incidence of developing dry eye (DE) disease during or after surgery due to reduced tear secretion and decreased tear film stability.^1^–^3^ In the early stage of DE, discomfort such as dryness, burning, pain, and foreign body sensation can seriously affect patient’s quality of life, lead to visual impairment, reading and driving difficulties, and significantly impact work and daily activities.^3^–^5^ Additionally, DE may also cause damage to the ocular surface tissue, leading to serious complications such as corneal ulcers, conjunctivitis, and corneal epithelial opacity.^5^,^6^

Currently, treatment of DE is focused on increasing eye moisture, promoting tear secretion, and stabilizing the tear film. Cyclosporine-A (CsA) and Diquafosol (DQS) are the two most popular types of topical anti-inflammatory and secretagogue agents. CsA is an immunosuppressive drug that reduces ocular surface inflammation and promotes tear secretion by inhibiting T cell activation and infiltration, while DQS is a P2Y2-receptor agonist that can provide deficient tear components and increase tear stability.^7^ However, there is relatively little comparative research on the use of CsA and DQS in the treatment of DE after CS. This study aimed to compare the efficacy of 0.05% CsA eye drops and 3% DQS that are administered in addition to the conventional treatment in CS patients with postoperative DE.

## METHODS

Clinical records of 123 patients with postoperative DE who were treated at the Fourth Hospital of Hebei Medical University from June 2022 to August 2023 were retrospectively analyzed. According to different treatment regimens, patients were divided into three groups: patients who received the routine treatment with tobramycin & dexamethasone eye drops combined with pranoprofen eye drops were included in the Conventional group, patients who received 3% DQS in addition to the conventional treatment were included in the DQS group, and patients who received 0.05% CsA eye drops (II) treatment on the basis of routine treatment were included in the CsA group.

### Ethical Approval:

This study was conducted in accordance with the principles of the Declaration of Helsinki and was performed after approval from the ethics committee (No. 2021KY328). June 2022.

### Inclusion criteria:

- After CS.
- Meets the diagnostic criteria for DE based on the “Chinese Dry Eye Expert Consensus: Immune Disease Related Dry Eye (2021)”.^8^
- All are single eye surgeries.
- Complete a one-month treatment cycle.
- The clinical data is completed.

### Exclusion criteria:

- Preoperative diagnosis of DE.
- Pregnant or lactating women.
- Severe heart, brain, liver, and kidney dysfunction.
- Mental illness or cognitive impairment.
- Patients with Diabetes.
- Patients with dry eyes caused by other ophthalmic diseases or other reasons
- Patients with a history of ophthalmic surgery or eye trauma.
- Patients with history of use of any drugs systemic and topical which can affect tear film, or long-term use of eye medication.

### Treatment regimens:

Conventional group: Patients received tobramycin & dexamethasone eye drops (Jiangxi Zhenshiming Pharmaceutical Co., Ltd., specification: 5 mL) one drop every 4-6 hours and received pranoprofen eye drops (Guangdong Zhongsheng Pharmaceutical Co., Ltd., specification: 5mL), four times per day, one drop per time.

### DQS group:

In addition to the conventional treatment, patients also received 3% Diquafosol Eye Drops (Qilu Pharmaceutical Co., Ltd., specification: 5ml: 150mg), one drop/time, 5-6 times/day.

### CsA group:

In addition to the conventional treatment, patients also received 0.05% CsA eye drops (II) (produced by Shenyang Xingqi Eye Medicine Co., Ltd., specification: 0.4ml: 0.2mg), one drop/time, two to three times/day.

All groups were treated for one month.

### Observation indicators and evaluation methods:

### Chief complaint score:

Patient’s visual fluctuations, visual fatigue, tingling, dryness, and redness were evaluated before and after the treatment. A scale of 1 to 5 was developed for assessing the severity of the symptoms, with higher scores indicating more severe symptoms.

### Tear meniscus height:

The Optovue RTVue Optical Coherence Tomography (OCT) system (Optove, USA, RTVue-100, axial resolution of 5μm, scanning rate of 26000 times/second) and CAM-L lens were used for inspection. Single line scanning mode was selected, with a scanning line length of 6mm, perpendicular to the lower eyelid margin, and placed at the six o’clock position of the cornea. When a stable tear stream image appeared, patient was instructed to blink once and a scan was performed for a total of three times. Random built-in measuring tool was used to measure the tear meniscus height, and the average of three measurements was taken.

### Conjunctival congestion score:

A large number of blood vessels in the conjunctiva of the eyeball, which are in a congested state and involve the bulbar conjunctiva, were rated as four points; Thickened blood vessels that affect 2/3 of the eyeball conjunctiva were rated as three points; Mild conjunctival vasodilation with a large quantity of blood vessels was rated as two points; A small amount of mild conjunctival vasodilation near the fornix was rated as one point; Absence of dilation or congestion in the blood vessels was rated as 0 points.

### Corneal fluorescein staining (FL) score^9^:

Severe staining was rated as three points; More than five stained spots was rated as two points; Five or less stained spots was rated as one point; No staining was rated as 0 points. Briefly, the cornea was divided into four equal parts, which were scored from 0 to 3 points according to the degree of staining. Finally, the total score of each part was added up, with the highest score being 12 points.

### Ocular surface disease index (OSDI) score:

A questionnaire that is used to discriminate between normal, mild to moderate, and severe DE.^10^ OSDI has a total of 12 questions, each rated at 0-4 points based on the patient’s perception of the severity of the symptom. Higher score indicates more severe symptom.

### Schirmer’s I test (SIt):

The SIt is used to evaluate aqueous tear production.^9^ For the test, patient was instructed to look straight ahead. One end of the measuring paper, bent by 5 mm, was applied into the inferior-temporal aspect of the conjunctival sac. Patient was guided to gently close their eyes for a trial time of five minutes, after which the wetted length in millimeters was measured.

### Tear film break-up time (TBUT):

TBUT is a method for determining the stability of the tear film and checking evaporative dry eye.^11^ For the procedure, patient was guided to sit with their gaze up, and 1% to 2% corneal fluorescein sodium solution was applied under the eyelid. Patient was instructed to blink quickly several times, taken into the dark room and positioned in front of the slit lamp. Patient’s position and the focus of the physician’s eyepiece were adjusted, and patient was guided to look forward without blinking. Stopwatch was used to record the time from blinking to the appearance of black spots, and the average value of three times in a row was recorded.

### Levels of serum inflammatory factors:

Serum levels of interleukin-6 (IL-6) and tumor necrosis factor alpha (TNF-α) were measured using enzyme-linked immunosorbent assay

### Cytokine levels in tears:

Levels of transforming growth factor 1 (TGF-β1) in collected tear fluid were detected by enzyme-linked immunosorbent assay, and levels of epidermal growth factor (EGF) were detected by radioimmunoassay.

### Statistical analysis:

SPSS 26 was used for all analyses. For count data, Chi square test or Fisher’s exact test were used for inter-group comparison. Quantitative data were represented by mean ± standard deviation, repeated measures ANOVA was used for inter group comparisons at multiple time points, LSD-t test was used for pairwise comparisons, and independent sample t-test was used for inter-group comparisons. *P*<0 05 indicates a statistically significant difference.

## RESULTS

A total of 123 patients (64 males and 59 females) were included in this study. Of them, 41 patients were included in the Conventional group, 42 patients were included in the DQS group, and 40 patients were included in the CsA group. There was no significant difference in gender, age, BMI, disease duration, and affected eye among the three groups (*P*>0.05) (Table-I).

**Table-I:** Comparison of baseline data among three groups of patients.

Baseline data	Conventional group (n=41)	Diquafosol group (n=42)	CsA group (n=40)	χ^2^/F	P
Gender (male/female)	25/16	17/25	22/18	3.702	0.157
Age (years)	62.98±7.85	61.648±7.16	63.45±6.62	0.692	0.502
BMI (kg/m²)	23.28±2.64	23.41±3.13	22.53±2.55	1.170	0.314
Disease duration (months)	12.418±3.61	11.62±3.15	12.58±3.21	0.974	0.380
Affected eye (left/right)	25/16	27/15	31/9	2.816	0.245

Chief complaint, conjunctival congestion, FL, and OSDI scores of the DQS group and the CsA group were significantly lower than those of the conventional group (*P*<0.05), while there was no statistically significant difference between the DQS group and the CsA group (*P*>0.05) Table-II Before the treatment, SIt and TBUT values were comparable in the three groups (*P*>0.05). After the treatment, values of SIt and TBUT in the DQS and the CsA groups were significantly higher than those in the Conventional group (*P*<0.05), while there was no statistically significant difference between the DQS group and CsA group (*P*>0.05) (Table-III).

**Table-II:** Comparison of chief complaint score, tear meniscus height, conjunctival congestion score, FL score, and OSDI score among three groups after the treatment.

Index	Conventional group (n=41)	DQS group (n=42)	CsA group (n=40)	F	P
Complaint symptom score	5.97±1.19	2.71±1.11^a^	2.80±1.22^a^	102.491	<0.001
Tear meniscus height	0.36±0.05	0.35±0.07^a^	0.37±0.06^a^	1.365	0.259
Conjunctival congestion score	2.83±0.38	1.05±0.22^a^	1.03±0.16^a^	606.947	<0.001
FL score	5.02±1.27	2.74±1.27^a^	2.68±1.25^a^	45.915	<0.001
OSDI score	2.95±0.22	1.38±0.66^a^	1.53±0.51^a^	124.756	<0.001

***Note:*** Compared with the conventional group,

a P<0.05.

**Table-III:** Comparison of SIt and TBUT levels among three groups of patients before and after treatment.

Group	SIt (mm/5 minute)		TBUT (second)	

	Before treatment	After treatment	Before treatment	After treatment
Conventional group	7.71±2.33	11.32±2.52^b^	6.49±2.35	11.51±2.74^b^
DQS group	7.90±2.15	15.94±2.84^ab^	6.86±2.48	16.74±2.56^ab^
CsA group	7.20±2.72	15.15±2.89^ab^	6.30±2.31	16.10±3.04^ab^
F	0.93	33.064	0.538	43.133
P	0.397	<0.001	0.560	<0.001

***Note:*** Compared with the conventional group, ^a^P<0.05; Compared with before treatment in this group,

b P<0.05.

Before the treatment, there was no statistically significant difference in the levels of serum inflammatory factors among the three groups (*P*>0.05). After the treatment, there was a marked decrease in the levels of IL-6 and TNF-α in the DQS group and the CsA group compared to the Conventional group (*P*<0.05). No statistically significant difference was detected between the DQS group and CsA group (*P*>0.05) (Table-IV).

**Table-IV:** Comparison of serum inflammatory factor levels before and after treatment in three groups.

Group	IL-6 (ng/L)		TNF-α (ng/L)	

	Before treatment	After treatment	Before treatment	After treatment
Conventional group (n=41)	121.61±14.79	56.93±5.27^b^	101.68±12.62	66.20±10.22^b^
DQS group (n=42)	124.30±12.45	31.68±4.56^ab^	103.98±11.46	36.02±6.34^ab^
CsA group (n=40)	125.47±13.81	33.69±6.60^ab^	106.34±14.16	37.31±7.27^ab^
*F*	0.848	264.997	1.349	181.944
*P*	0.431	<0.001	0.263	<0.001

***Note:*** Compared with the conventional group, ^a^P<0.05; Compared with before treatment in this group,

b P<0.05

Before the treatment, the cytokine levels in the tears of the three groups were similar (*P*>0.05). However, after the treatment, levels of EGF and TGF-β1 in the DQS group and CsA group became significantly higher compared to those of the Conventional group (*P*<0.05). There was no statistically significant difference in the post-procedure levels of cytokines between the DQS and the CsA group (*P*>0.05) (Table-V).

**Table-V:** Comparison of cytokine levels in tears before and after treatment among three groups

Group	EGF (ng/mL)		TGF-β1 (pg/mL)	

	Before treatment	After treatment	Before treatment	After treatment
Conventional group (n=41)	2.65±0.29	3.36±0.41^b^	35.71±4.80	40.48±6.15^b^
DQS group(n=42)	2.55±0.51	5.58±0.51^ab^	36.60±5.53	45.35±6.46^ab^
CsA group (n=40)	2.49±0.33	5.77±0.76^ab^	34.57±5.57	47.00±7.89^ab^
*F*	1.852	218.898	1.496	9.94
*P*	0.161	<0.001	0.228	<0.001

***Note:*** Compared with the conventional group, ^a^P<0.05; Compared with before treatment in this group,

b P<0.05.

## DISCUSSION

Our result showed a marked improvement in main DE indicators, such as the chief complaint score, conjunctival congestion score, FL score, and OSDI score, in CS patients who received 0.05% CsA eye drops (II) or 3% DQS treatment compared to patients who were treated by the conventional treatment. This indicates that both 0.05% CsA and 3% DQS have a certain effect in alleviating DE symptoms, which is consistent with the findings by Lee et al.^12^ It has been reported that 0.05% CsA eye drops (II) can regulate the lipid composition in the tears of patients with DE, improve tear film stability, and reduce ocular surface inflammation.^13^ We may speculate that CsA can stimulate the recovery of tear gland function, help increase tear production, alleviate eye inflammation, reduce the damage of inflammatory reactions to the surface of the eye, thereby promoting tear secretion and improving DE.^14^,^15^

Meanwhile, Miura et al.^16^ found that postoperative use of 3% DQS in eye patients can promote tear secretion and improve postoperative DE. Our results further confirm these observations. Studies show in CS patients, the associated surgical stimulation and damage to the eye tissue suppress activity of the lacrimal gland, leading to reduced tear secretion and the formation of DE.^2^,^17^ CsA can stimulate the recovery of tear gland function, improve tear stability, regulate the adhesion and secretion of corneal surface cells, increase SIt, and improve the condition of eye tissue by inhibiting inflammatory reactions and immune-mediated damage.^13^,^14^ Similarly, 3% DQS can regulate immune response, stimulate tear gland secretion function, increase corneal epithelial adhesion, improve tear stability, restore corneal epithelial barrier function, increase tear production, reduce water evaporation, and prolong tear film rupture time.^15^,^16^

The results of this study also showed that 0.05% CsA eye drops(II) or 3% DQS treatment were associated with significant improvement in the levels of IL-6, TNF-α, EGF, and TGF-β1. This suggests that DQS and CsA have a certain impact on the levels of serum inflammatory factors and cytokines in tear fluid. It is possible that CsA may have multiple effects. CsA was shown to stimulate the production of epidermal growth factor (EGF) and promote the proliferation and repair of corneal epithelial cells,^18^ and EGF acts on promoting cell division and proliferation, accelerating wound healing and repair, thereby improving the function of the corneal epithelial barrier.^17^,^19^ Moreover, CsA reduces corneal fibrosis and scar formation by inhibiting the production and release of TGF-β1, thereby maintaining corneal transparency and normal structure.^20^,^21^ Additionally, CsA can balance the activity of the immune system, alleviate inflammatory reactions, and lower serum IL-6 and TNF-α levels in patients with DE after CS.^16^,^22^ Studies also show that CsA can regulate secretion, and activity of cytokines in tears, and regulate their interaction. It can inhibit the release of inflammatory mediators and signal transduction, reduce the damage of inflammatory reactions to ocular surface tissue, and promote the restoration of cytokine balance.^23^,^24^

This study also showed that both 3% DQS and 0.05% CsA eye drops (II) are associated with marked beneficial effect in CS patients with DE. Our results suggest that for patients with DE after CS, treatment regimen should be selected based on their actual situation. Patients should be guided to adhere to standardized use of the medications under supervision to further improve clinical efficacy.

### Limitations:

Firstly, this is a single center retrospective study. Secondly, none of the three groups were randomly assigned, and baseline information may be imbalanced and biased. Thirdly, relevant indicators may have been influenced by human or technical factors. Fourthly, after regular treatment, patients were not followed-up for an extended period of time, and the frequency of daily use of eye drops was not monitored, despite emphasizing their importance to patients. Therefore, it cannot be ruled out that the overall therapeutic effect may have been reduced due to the variability in patient compliance. Further research is needed to clarify the mechanisms of 3% DQS and 0.05% CsA eye drops action (II).

## CONCLUSION

Compared to the conventional treatment alone, 3% dexamethasone or 0.05% CsA eye drops (II) in addition to the conventional treatment can improve the DE treatment effect, and alleviate conjunctival congestion and inflammatory reactions in patients with DE after CS.

### Authors’ contributions:

**HL:** Conceived, designed the study and involved in the writing of the manuscript and is responsible for the integrity of the study.

**SZ, CS, PC, MM and YH:** Collected the data and performed the analysis and critical Review.

All authors have read and approved the final manuscript.
